# Impact of downstream effects of glucocorticoid receptor dysfunction on organ function in critical illness-associated systemic inflammation

**DOI:** 10.1186/s40635-020-00325-z

**Published:** 2020-12-18

**Authors:** Martin Wepler, Jonathan M. Preuss, Tamara Merz, Oscar McCook, Peter Radermacher, Jan P. Tuckermann, Sabine Vettorazzi

**Affiliations:** 1 Institute for Anesthesiological Pathophysiology and Process Engineering, University Hospital Ulm, Ulm, Germany; 2 Department of Anesthesia, University Hospital Ulm, Ulm, Germany; 3 Institute of Comparative Molecular Endocrinology (CME), Ulm University, Ulm, Germany

**Keywords:** Glucocorticoid receptor, Dysfunction, Monomer, Dimer, Systemic inflammation, Mouse models, Organ function

## Abstract

Glucocorticoids (GCs) are stress hormones that regulate developmental and physiological processes and are among the most potent anti-inflammatory drugs to suppress chronic and acute inflammation. GCs act through the glucocorticoid receptor (GR), a ubiquitously expressed ligand-activated transcription factor, which translocates into the nucleus and can act via two different modes, as a GR monomer or as a GR dimer. These two modes of action are not clearly differentiated in practice and may lead to completely different therapeutic outcomes. Detailed aspects of GR mechanisms are often not taken into account when GCs are used in different clinical scenarios. Patients, with critical illness-related corticosteroid insufficiency, treated with natural or synthetic GCs are still missing a clearly defined therapeutic strategy. This review discusses the different modes of GR function and its importance on organ function in vivo.

## Background

Glucocorticoids (GCs) belong to the steroid hormones and are derived from cholesterol through different intermediates. They are released by the adrenal glands, in a circadian rhythm, and play a crucial role in the adult physiology: immune function, glucose metabolism, and blood pressure regulation. This GC homeostasis (circadian rhythm) could be challenged and affected by acute and chronic stress and systemic inflammation. The GCs have multifaceted actions and therefore an impact on metabolism, bone, and immune system. The GCs—as anti-inflammatory and immunosuppressant drug—are already used since 1948 by Philip Hench and Edward Kendall to treat patients with rheumatoid arthritis and efficiently reduce the inflammatory effects [1]. This was awarded with the Nobel Prize in 1950 [2]. Until present, synthetic GCs are indispensable for the treatment of inflammatory diseases.

## Importance of glucocorticoids in critical illness

**Fig. 1:**
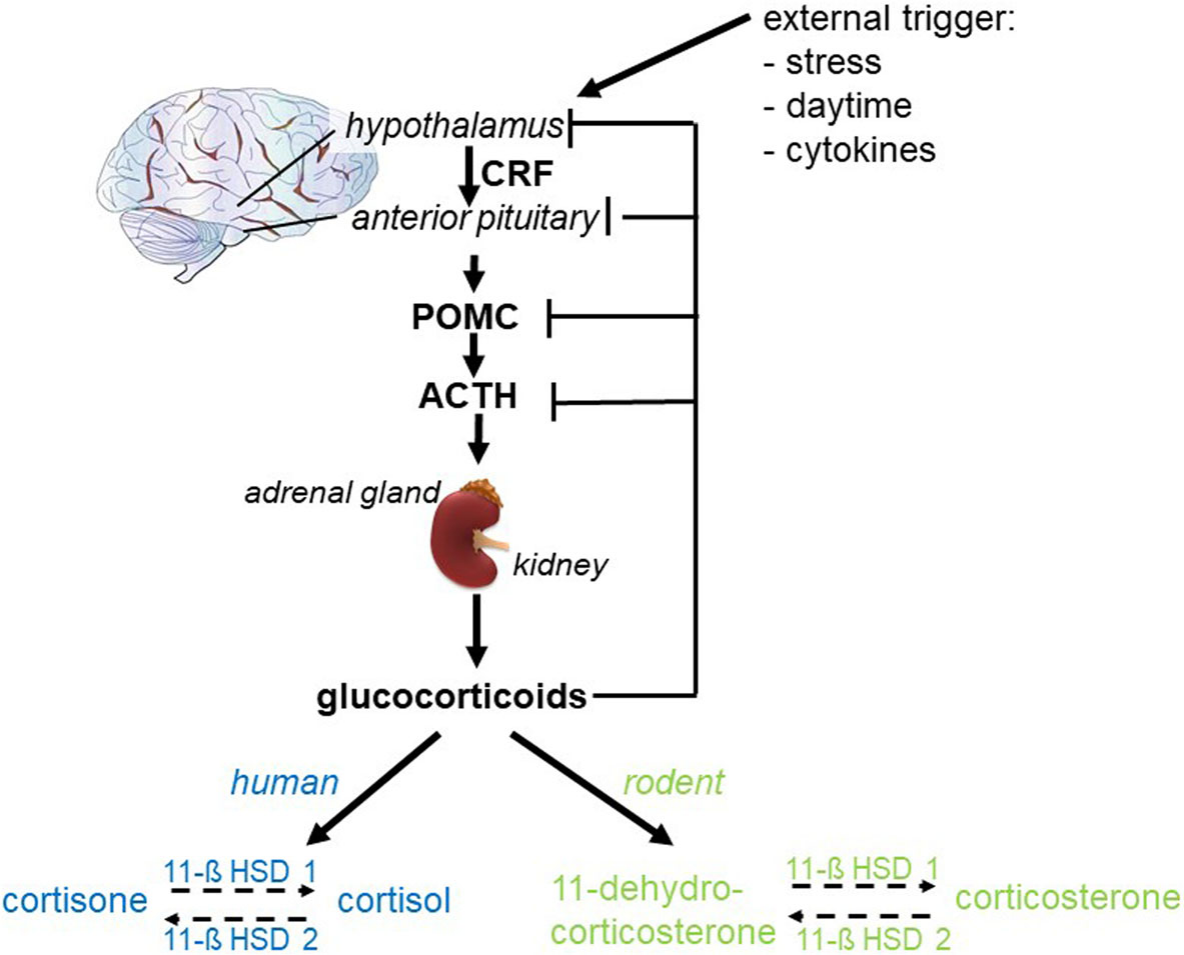
After the activation of the hypothalamus by external triggers, corticotropin-releasing factor (CRF) acts on the anterior pituitary to release proopiomelanocortin (POMC), which in turn is a proteolytic cleaved in adrenocorticotropic hormone (ACTH) and activates the production of enzymes for the corticosteroid synthesis in the adrenal glands. Glucocorticoids act in a negative feedback mechanism to regulate their own production. In humans, cortisone is converted by 11-β-hydroxysteroid-dehydrogenase-1 (11β-HSD1) to cortisol and vice versa by 11β-HSD2. In rodents, the 11-dehydrocorticosterone is converted to corticosterone and vice versa

Critical illness represents severe acute stress and is therefore often accompanied by high levels of GCs (cortisol in humans and corticosterone in rodents, Fig. 1). This observation has traditionally been attributed to stress-induced activation of the hypothalamic–pituitary–adrenal (HPA) axis and the subsquently increased corticotropin-driven GC production (Fig. 1, [3]). Furthermore, suppressed expression and activity of cortisol-metabolizing enzymes lead to a reduced cortisol breakdown, which also contributes to hypercortisolemia and, hence, adrenocorticotropin suppression [4]. However, in patients with critical illness, the systemic availability of cortisol may be not high enough to face the stress induced by the illness and, together with the hypercortisolemia-induced corticotropin suppression, present as “critical-illness-related corticosteroid insufficiency” (CIRCI). Besides, patients with CIRCI often present “corticosteroid resistance”, which indicates CIRCI as a relative adrenal failure, because the cortisol levels are high but do not induce their regular effects within this corticosteroid resistance [5, 6]. In critically ill patients with sepsis, the excessive cytokine production may further suppress later adrenocorticotropin hormone synthesis [7, 8] and the cortisol response to exogenous adrenocorticotropin hormones [9, 10], thereby aggravate CIRCI. However, there is still an ongoing discussion about GC therapy in critical illness, especially in acute respiratory distress syndrome (ARDS) and sepsis. The lack of clarity of the effects of GC treatment in critical illness may also be related to different effects and states of glucocortiocid receptor (GR) function, because GCs mainly mediate their effects by binding to the GR. This review will focus on the role of GR fuction in criticall ilness-associated systemic inflammation and the downstream effects on organ function.

## The endogenous glucocorticoid biosynthesis and importance in homeostasis

Glucocorticoids (GCs) belong to the steroid hormones, which are derived from cholesterol through different intermediates like progesterone. GCs have a crucial role in physiology (immune function, glucose metabolism, and blood pressure regulation), but also during development: GCs are important for the differentiation of chromaffin cells and bone integrity [11–14] and, moreover, for lung maturation [13].

The synthesis of steroids takes place in the mitochondrium and the endoplasmic reticulum of steroid-synthesizing cells. The rate-limiting enzymes of the steroid synthesis are cholesterol esterase, steroidogenic acute regulatory protein (Star), and cytochrome P450 [15]. GCs are produced in the adrenal glands, in the *zonae fasciculata* which is the intermediate part of the adrenal glands. GC production is mediated by a hierarchical hormonal signaling that is composed of the hypothalamus, the pituitary, and the adrenal, designated as the HPA axis. Upon input of centers that maintain the circadian rhythm, the occurrence of acute stress, or systemic inflammation, the hypothalamus releases the corticotropin-releasing factor (CRF). CRF acts on the adenohypophysis resulting in the synthesis of proopiomelanocortin (POMC) [16]. POMC is proteolytically cleaved into adrenocorticotropic hormone (ACTH) and activates the production of enzymes for the corticosteroid synthesis in the adrenal cortex (Fig. 1). The concentration of GCs is regulated by a negative feedback mechanism: high levels of GCs suppress POMC and CRF expression and thereby shut down the central synthesis of GCs. This is the underlying mechanism for the ultradian and diurnal rhythm of corticosterone in rodents and cortisol in humans. Once delivered into the blood, almost 90% of the hydrophobic released GCs are bound, transported, distributed, and released into the cell with the help of corticosteroid-binding globulin (CBG), also known as transcortin [17]. Within the cells, the 11 β-hydroxysteroid dehydrogenase 1 and 2 (11β-HSD1 and 11β-HSD2) control the bioavailability of the GCs. The 11β-HSD1 converts the inactive into the active form (human: cortisone into cortisol; rodents: 11-dehydrocorticosterone into corticosterone). Conversely, the active form (human: cortisol; rodents: corticosterone) is oxidized by 11β-HSD2 to the inactive form (Fig. 1, [18]). Due to the differential expression of 11β-HSD1 (which amplifies GC concentrations) or/and 11β-HSD2 (which reduces GC concentrations), GC sensitivity can influence a metabolic syndrome caused by obesity and/or insulin resistance [19, 20].

## Molecular mechanims of GC action: transcactivation transrepression

**Fig. 2:**
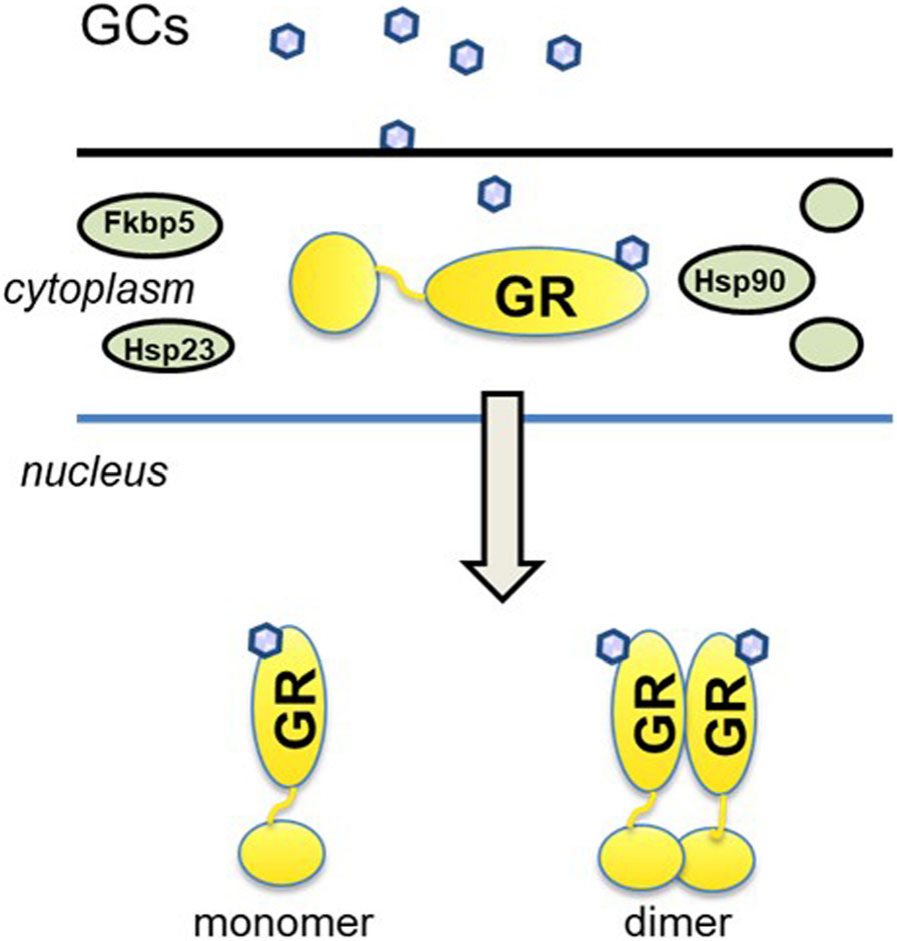
After binding of the glucocorticoids (GCs are shown as small ellipses) as ligands to the glucocorticoid receptor GR, the immunophillins (Fkbp51, Fkbp52), heat shock proteins (Hsp70, Hsp90, p23), and chaperonic molecules dissociate and the GR translocates in the nucleus. In the nucleus, the ligand-bound GR can either act as a monomer or a homodimer

GCs act via two receptors, the high-affinity mineralocorticoid receptor (MR) and the low-affinity glucocorticoid receptor (GR). However, MR expression seems to be more restricted to distinct tissues, in some of which GCs are inactivated by 11β-HSD2. Since the GR is more widespread, the majority of GC action is indeed mediated by the GR. The GR is a nuclear receptor that resides in the cytoplasm complexed with immunophilins (Fkbp5), heat shock proteins (Hsp70, Hsp90, p23), and chaperone molecules in the absence of ligand [21–23]. After binding of the ligand (e.g., GCs) to the GR, a conformational change together with the Hsp allows a proper folding of the GR. The major fraction of the GR molecules translocates into the nucleus after binding to the ligand and directly acts on gene regulation. To this end, the immunophilins and Hsps must dissociate to allow interaction of the GR with the protein import machinery. In the nucleus, the ligand-bound GR can either act as a single molecule (monomer) or as a homodimer (2 GR molecules, Fig. 2).

As a dimer, the GR binds to GR binding sites (GBS). The classical sequence motif of the GBS is the GR response element (GRE) that is comprised of a palindromic DNA sequence (GGAACAnnnTGTTCT), which is separated by a 3-base pair spacer (n), but also display a certain degree of degeneration of the consensus sequence [24, 25]. The DNA-bound GR homodimer recruits co-regulatory molecules by its N-terminal and C-terminal transactivation functions that lead to chromatin remodeling, a prerequisite to induce transcription of GC-regulated genes. Genome-wide analyses revealed that tissue-specific transcription factors poise the chromatin for tissue-selective GR binding to GBS that lead to a tissue-specific hormone response [26–30].

The monomer GR either directly binds to DNA at the so-called half-GREs (1/2 GRE) that contain one half of the palindromic sequence of the classical GRE or, alternatively, interacts with other transcription factors bound to their responsive elements [26, 31]. Among these, many were identified as transcription factors involved in cytokine regulation and regulation of other pro-inflammatory mediators. Of note, just recently, the GR was described to act as a tetramer, but the significance and physiological relevance of this finding still needs further investigation [32]. This shows that the GR biology is an important component and mediator of the GC effects that must be taken into consideration.

## The (almost) failed question of dissociating GR ligands

For GC therapy, a fundamental question is how to differentiate side effects from beneficial effects, in other words, to define therapeutic windows for immune modulatory functions, to allow a safer GC therapy, by either reducing side effects or increasing the efficacy. The discovery that GR acts as a dimer or as a monomer provoked great hopes to develop dissociating ligands that could promote beneficial effects while avoiding GR-mediated side effects [33]. In the pre-genome era, when only a limited set of GR target genes were known, (i) the bona fide GR dimer-dependent target genes were genes involved in glucoconeogenesis in the liver and (ii) the GR monomer AP-1-dependent matrix metalloproteases and NF-kB-dependent cytokine genes, which were repressed by GCs. With this limited knowledge about GR-mediated gene regulation, drug screening programs for dissociating GR ligands were initiated that aimed to disrupt GR dimerization, but leaving GR monomer function intact with the goal to enable repression of cytokines and to avoid negative effects on glucose metabolism. However, only a few of these compounds made it into preclinical trails for topical application [34].

The last 15 years of research have revealed that for full efficacy of GCs, the GR dimer-dependent transactivation of anti-inflammatory or immune modulatory genes are necessary. In addition, the paradigm that GCs just suppress inflammation is changing to a concept that the immune system is modulated into an active mode of resolution of inflammation. Indeed, GCs are potent agents that induce the activation of anti-inflammatory monocytes/macrophages [35–37]. Important insights were also provided by a mouse knock-in model, which impaired GR dimerization, the so called GR^dim^ mouse, a tool that is availbale to investigate the impact of the GR dimerization in vivo [38].

## GR mutant mouse models and GC therapy

**Fig. 3:**
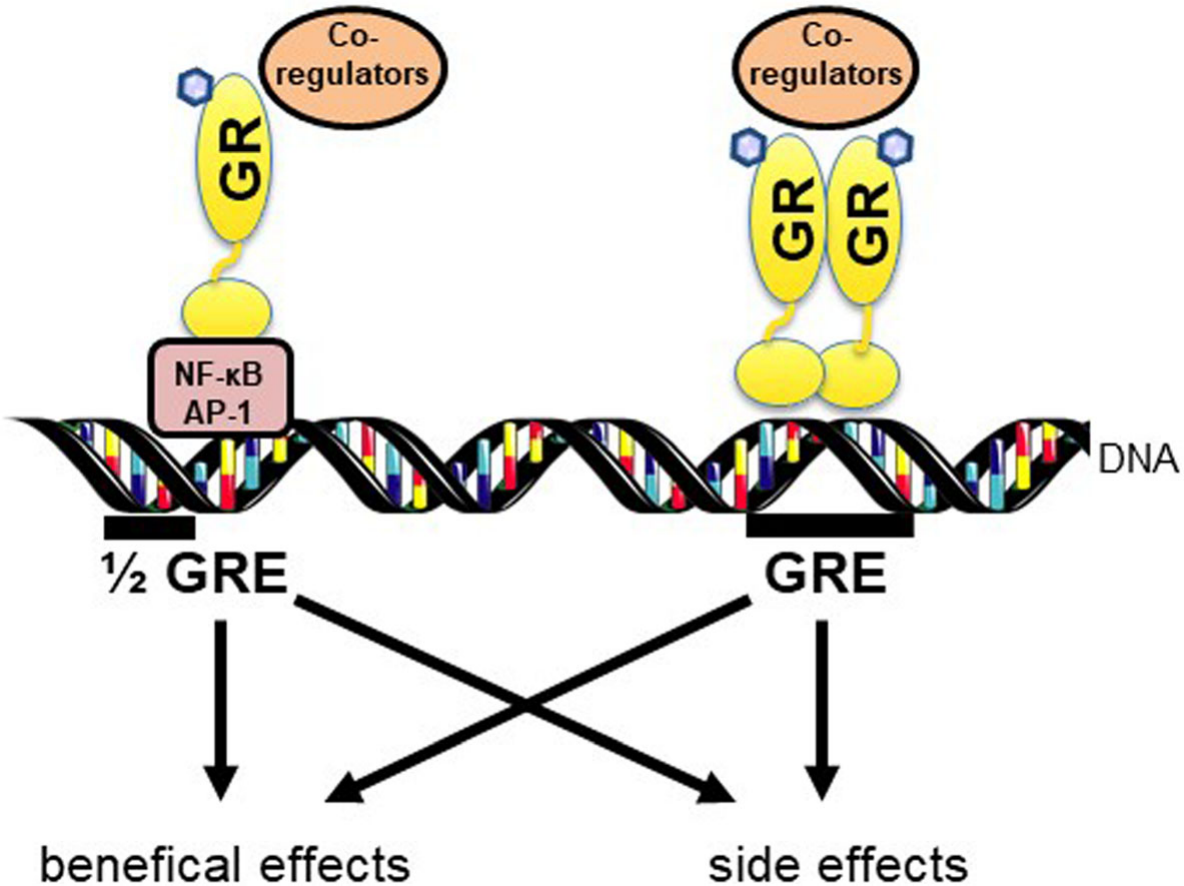
The classical dogma that the glucocorticoid receptor (GR) monomer by interaction with pro-inflammatory transcription factors with AP-1, NF-κB, or half glucocorticoid response elements (1/2 GRE) mediate beneficial effects while the dimer mediates side effects by direct binding to GREs does not hold true. The GR dimer is also important to mediate beneficial effects and the monomer side effects

GR^dim^ mice have a point mutation in the DNA binding domain of the GR and therefore reduced GR homodimerisation and, subsquently, reduced GRE binding capacity [26, 38, 39]. In contrast to the complete knockout mice [13], these GR^dim^ mice survive until adulthood, which allows their study in disease models. In an irritant skin inflammation model induced by phorbolester or experimental autoimmune encephalomyelitis, a mouse model for multiple sclerosis, GR^dim^ mice respond effectively to GC treatment, indicating that GR monomer action might be sufficient to reduce inflammation [40, 41]. In contrast, in most other inflammatory models tested, like lipopolysaccharide (LPS)-, tumor necrosis factor α (TNFα)-, and cecal ligation and puncture (CLP)-induced systemic inflammation, mice with impaired GR dimerization (GR^dim^) were highly susceptible to inflammation, had elevated cytokines, dysregulated metabolic pathways, and impaired thermoregulation [42]. This demonstrates that the dimerization of the GR is crucial for immune modulatory actions of endogenous GCs. Furthermore, GR^dim^ mice treated with exogenous GCs showed impaired anti-inflammatory response in a variety of inflammatory models: acute lung injury [43], rheumatoid arthritis [44], contact allergy [45], and allergic airway inflammation [46]. All these models again emphasize that the GC (endogenous or exogenous) activated GR dimer has substantial impact on the modulation of inflammation. GC activation of the GR was hypothesized to counteract inflammation. However, in inflammatory mouse models, it was shown that the GR dimer in synergy with pro-inflammatory signaling induced crucial genes that led to the resolution of inflammation: sphingosinekinase 1 [43], metallothionein1 and 2 [47, 48], and Serpin A3 [49]. This reveals that both anti-inflammatory and pro-inflammatory actions work together to resolve inflammation. Resistance to GC treatment in GR SUMOylation (a posttranslational modification) mutant mice during skin inflammation due to reduced co-repressor recruitment further contribute to the understanding of GC binding to GR actions [50, 51]. Furthermore, mice with a mutation in the most active isoform of the GR (C3), where the GR is activated by endogenous GCs through the activation of the HPA axis during endotoxemia, are hypersensitive to LPS-induced systemic inflammation [52]. Thus, by studying mutant mice with an impaired GR dimerization in a variety of inflammatory models, novel molecular mechanims of the GR were discovered, which shows that the old classical dogma “GR monomer mediates only beneficial effects while the dimer mediates only side effects” does not hold true (Fig. 3). This explains to a major extent why the use of selective glucocorticoid receptor agonists (SEGRAs) as dissociating ligands failed in the clinics.

One way to improve GC therapy would be to identify the GC-mediated GR action in specific cell types that are necessary and sufficient for a whole body response. Valuable insights have been gained from cell type-specific conditional GR knockout mice, targeting the GR in various cell types, like myeloid cells and brain, muscle, or bone cells. We will mainly focus here on the GR-targeted deletion with the help of the Cre/loxP system in immune cells and impact during inflammation. Deletion of the GR in myeloid cells (macrophages, monocytes, and granulocytes) was achieved by crossing the Lysozyme 2 Cre recombinase (LysMCre) knock-in mice that were crossed with mice carrying flanking loxP alleles (GRflox) [45]. This led to an almost untouched GR expression, except in cells of the myeloid lineage, in which the Lyz2 gene was active, and cre recombinase recombined the GRflox locus to a GR knockout allele. In vivo experiments with GR^LysMCre^ mice revealed that the GC activation of GR is crucial in LPS-induced systemic inflammation [53, 54], dextran sodium sulfate (DSS)-induced colitis [55], and myocardial infarction [56] to reduce inflammation. The exogenous GC treatment is mediated through the GR in myeloid cells in models of acute lung injury [43] and contact hypersensitivity [45] to mediate anti-inflammatory effects. All these studies show that the GC activation of the GR in myeloid cells is of major importance for the immune modulatory outcome during inflammation. Intriguingly, not only is the GC activation/binding of the GR in immune cells crucial for a proper response, as discussed above, but even in non-immune cells (such as fibroblasts), the GC binding the GR indirectly triggers the resolution of inflammation by influencing immune cell polarization and, hence, outcome during inflammation [44, 57].

In the following sections, we will focus on the effects of impaired GR dimerization in mutant mice (GR^dim^ mice) and its impact on organ function during critical illness-associated systemic inflammation to demonstrate the impact of a functional GR that is activated by endogenous GCs.

## Effects of an impairment of GR dimerization on organ function in different mouse models

**Fig. 4:**
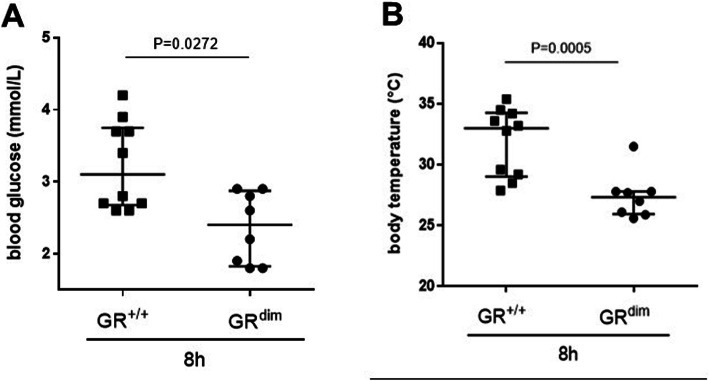
**a** Blood glucose levels and **b** body temperature in GR^dim^ and GR^+/+^ mice 8 h after LPS challenge without any hemodynamic or ventilatory support nor temperature control. Data is presented as median and interquartile range

The effects of systemic inflammation, i.e., resuscitated CLP-induced septic shock, on glucose metabolism in mice have been described previously [58]. Interesting preliminary results with LPS-induced systemic inflammation and the blood glucose levels in GR^dim^ mice led to additional studies (unpublished). When GR^dim^ [38] and wild-type contol mice (GR^+/+^) challenged with LPS without subsquent hemodynamic monitoring, ventilatory support, nor temperature control, the GR^dim^ mice exhibited low glycemic levels, which coincides with severe hypothermia in contrast to GR^+/+^ (Fig. 4a). Reduced core temperature and malaise was already described in a previous study of GR^dim^ LPS-induced inflammation without intensiv care management (Fig. 4b, [59]). In order to study the effects of LPS challenge in GR^dim^ mice and control for hemodynamics, lung function, and temperature, the mice were placed in a mouse intensive care unit (MICU). Although the focus was to study the effects of core temperature and the relation to blood glucose levels in this setup, it was detected that the GR dimer significantly contributed to hemodynamic instability during LPS-induced inflammation. The GR^dim^ mice, after the LPS challenge, required significantly higher doses of norepinephrine (NA) to maintain mean arterial pressure above 55 mmHg compared to GR^+/+^ mice [60]. These results indicate an important role for the GR dimer in regulating hemodynamic stability in LPS-induced systemic inflammation. In septic shock patients with systemic inflammation and severe hemodynamic instability, supplementation of hydrocortisone led to increased hemodynamic stability and a reduced need for vasopressors [61]. This beneficial effect of GCs on hemodynamic stability in severe septic shock in patients might be explained by stimulation of endothelial GRs [62] and an attenuation of the cytokine-mediated downregulation of vasoconstrictive receptors, such as α_1_-adrenergic receptors or the V_1A_ receptor [63]. Goodwin et al. studied mice with an endothelial-specific GR deletion and showed significantly more hemodynamic instability, which was accompanied by higher nitric oxide (NO) levels compared with controls [62]. It is well established that NO plays an important pathophysiological role in sepsis with both, direct and indirect deleterious as well as beneficial effects [64]. In patients, NO has well-known vasodilatory effects [65]. Thus, the higher NO levels in mice with an endothelial cell-specific GR deletion as reported by Goodwin most likely contributed to the hemodynamic instability in these animals. Although vascular-specific effects of an ubiquitous impairment of GR dimerization have not been examined while studying GR^dim^ mice after LPS-challenge, it is likely that the increased hemodynamic instability in GR^dim^ mice has been, at least in part, meditated via a NO-induced vasodilation [62]. In summary, these results demonstrate an important role for endogenous GCs mediated GR dimerization in maintaining hemodynamic stability during experimental systemic inflammation induced by LPS, with mechanisms other than solely endothelial GR function.

It is well-accepted clinical practice to treat asthma and chronic obstructive pulmonary disease (COPD) with synthetic GCs to reduce local and systemic inflammation [66–68]. Although approximately 20% of patients with ARDS receive synthetic GCs [69], there is no real evidence yet of its beneficial effects. Clearly, synthetic GCs may accelerate the resolution of respiratory failure, but their side effects have to be taken into account. Furthermore, there is an increased risk of mortality in patients when synthetic GCs are used > 14 days after the onset of ARDS [70]. Therefore, GCs should probably not be initiated after 2 weeks of the onset of ARDS because of the uncertain risk-to-benefit ratio. On the other hand, GCs inhibit fibroblast proliferation and collagen deposition, providing the rationale for synthetic GC treatment in non-resolving ARDS with the goal of preventing progression to fibroproliferative changes later in the course of the disease [71, 72].

The lack of clarity of the effects of GC treatment in lung injury may also be related to different effects and biology of GR function. A mouse model with a total GR knockout results in lethality shortly after birth due to respiratory failure, elevated ACTH level, elevated corticosterone levels, and reduced expression of gluconeogenetic enzymes [13]. Furthermore, lung function is in part maintained through the GR in airway epithelial cells [73] and mesenchymal GR facilitates the development of the respiratory system in mice [74]. This emphasizes that endogenous GCs acting through the GR are critical for maturation and development of lung function.

**Fig. 5:**
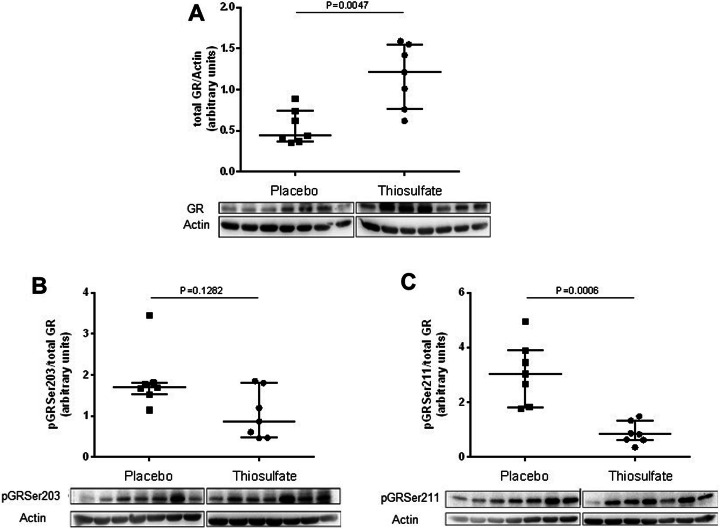
**a** Protein expression of the total glucocorticoid receptor (GR). **b** Protein expression of phosphorylated GR at serine 201 (pGRser203) and **c** phosphorylated GR at serine 211 (pGRser211) in lung tissue. **a**, **b**, and **c** are normalized to actin as loading control and thereafter to total GR. In **a**, **b**, and **c**, the lung tissue was analyzed from swine resuscitated from hemorrhagic shock and treated with sodium thiosulfate or placebo for the first 24 h of resuscitation after hemorrhagic shock [78]. Data is presented as median and interquartile range

In general, GR downregulation is reported to be associated with organ dysfunction in humans and different animal models, in particular in the liver [75], the heart [76], and the lung [77]. Vice versa, in swine with pre-existing coronary artery disease that underwent hemorrhagic shock (HS) and resuscitation, treatment with sodium thiosulfate (Na_2_S_2_O_3_) during the first 24 h of resuscitation attenuated the impairment of lung mechanics and gas exchange, which coincided with higher lung tissue GR expression (as assessed by western blotting and immunohistochemistry, Fig. 5a, [78]). Existing studies so far showed that the GR is transcriptionally more active when phosphorylated on Serine 211 (pSer211), in part due to a conformational change and increased recruitment to GRE-containing promoters [79]. The GR phosphorylation site Serine 203 (pSer203) is contained within the cytoplasmic fraction of the cell and fails to bind GRE-containing promoters, suggesting that pGRSer203 is a transcriptionally inactive form of the GR [79]. Therefore, two different phosphorylation sites of the GR (Serine 211 and 203) were investigated in lung tissue of swine with pre-existing coronary artery disease that underwent HS and resuscitation. While pGRSer203 remained unaltered (Fig. 5b), GRSer211 was significantly decreased in lung tissue of thiosulfate-treated animals (Fig. 5), suggesting a less active GR signaling. However, for GR target genes like glucocorticoid-induced leucine zipper (GILZ), mitogen-activated protein kinase phosphatase 1 (MKP1), or sphingosine kinase 1 (SPHK1, known to resolve the inflammatory response), similar expression were found in the lungs of thiosulfate-treated animals. This suggests that the Serine 211 phosphorylation site of the GR might not always indicate a higher activation level of the GR at this later time point (72 h after resuscitation) as it was already shown in neuronal cells [79].

## Conclusion

Basic research studies have shown that impaired glucocorticoid receptor (GR) dimerization increases mortality in lipopolysaccharide (LPS) and cecal-ligation-and-puncture (CLP)-induced inflammation and aggravates circulatory and pulmonary dysfunction after LPS-induced systemic inflammation. Attenuating GR dimerization results in resistance to exogenous glucocorticoids (GCs) to ameliorate acute lung injury (ALI). These results may indicate a role of the GR dimer to affect organ function in states of systemic inflammation and reveal some crucial immunmodulatory target genes. In addition, with the help of mutant mouse models lacking the GR in different cells and/or tissues, the importance of GR action may be dissceted to reveal basic mechanisms.

## Data Availability

Data sharing is not applicable to this article as no datasets were generated or analyzed during the current study.

## Abbreviations

11β-HSD1: 11 β-hydroxysteroid dehydrogenase 1
11β-HSD2: 11 β-hydroxysteroid dehydrogenase 2
ACTH: Adrenocorticotropic hormone
ALI: Acute lung injury
ARDS: Acute respiratory distress syndrome
CBG: Corticosteroid-binding globulin
CIRCI: Critical illness-related corticosteroid insufficiency
CLP: Cecal ligation and puncture
COPD: Chronic obstructive pulmonary disease
CRF: Corticotropin-releasing factor
DSS: Dextran sodium sulfate
GBS: Glucocorticoid receptor binding sites
GCs: Glucocorticoids
GILZ: Glucocorticoid-induced leucine zipper
GR: Glucocorticoid receptor
GR^dim^: Glucocorticoid receptor dimerization
GRE: Glucocorticoid receptor response element
HPA: Hypothalamic–pituitary–adrenal
HS: Hemorrhagic shock
Hsp: Heat shock proteins
LPS: Lipopolysaccharide
MICU: Mouse intensive care unit
MKP1: Mitogen-activated protein kinase phosphatase 1
MR: Mineralocorticoid receptor
NA: Norepinephrine
Na_2_S_2_O_3_: Sodium thiosulfate
NO: Nitric oxide
POMC: Proopiomelanocortin
SEGRAs: Selective glucocorticoid receptor agonists
SPHK1: Sphingosine kinase 1
TNFα: Tumor necrosis factor α
